# Recent Advances in Carbon Nanodots: A Promising Nanomaterial for Biomedical Applications

**DOI:** 10.3390/ijms22136786

**Published:** 2021-06-24

**Authors:** Safeera Khan, Andrew Dunphy, Mmesoma S. Anike, Sarah Belperain, Kamal Patel, Norman H. L. Chiu, Zhenquan Jia

**Affiliations:** 1 Department of Biology, University of North Carolina at Greensboro, Greensboro, NC 27412, USA; skhan91@midwestern.edu (S.K.); adunphy@uncc.edu (A.D.); msanike@uncg.edu (M.S.A.); srbelper@uncg.edu (S.B.); kkpatel3@uncg.edu (K.P.); 2 Department of Chemistry and Biochemistry, University of North Carolina at Greensboro, Greensboro, NC 27412, USA; nhchiu@uncg.edu; 3 Department of Nanoscience, Joint School of Nanoscience and Nanoengineering, University of North Carolina at Greensboro, Greensboro, NC 27401, USA

**Keywords:** carbon nanodots, nanomaterials, biomedicine, ROS scavenging mechanism

## Abstract

Carbon nanodots (CNDs) are an emerging class of nanomaterials and have generated much interest in the field of biomedicine by way of unique properties, such as superior biocompatibility, stability, excellent photoluminescence, simple green synthesis, and easy surface modification. CNDs have been featured in a host of applications, including bioimaging, biosensing, and therapy. In this review, we summarize the latest research progress of CNDs and discuss key advances in our comprehension of CNDs and their potential as biomedical tools. We highlighted the recent developments in the understanding of the functional tailoring of CNDs by modifying dopants and surface molecules, which have yielded a deeper understanding of their antioxidant behavior and mechanisms of action. The increasing amount of in vitro research regarding CNDs has also spawned interest in in vivo practices. Chief among them, we discuss the emergence of research analyzing CNDs as useful therapeutic agents in various disease states. Each subject is debated with reflection on future studies that may further our grasp of CNDs.

## 1. Introduction

**Figure 1:**
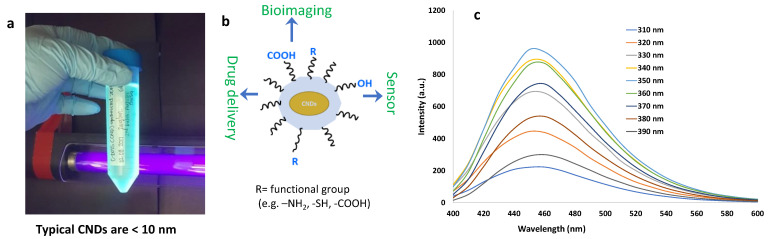
(**a**) CNDs fluorescence in a tube; (**b**) functional groups and applications of CNDs; (**c**) fluorescence spectrum with an emission peak of ~450 nm and excitation wavelengths from 400 to 600 nm.

Carbon nanodots (CNDs) are a novel addition to the family of nanomaterials, with exceptional biocompatibility, stability, fluorescence (Figure 1), and photoluminescence [1,2,3,4]. The typical CNDs are <10 nm and have a lattice spacing with a chemical composition of carbon, oxygen, and surface functional groups (Figure 1). X-ray photoelectron spectroscopy (XPS) can determine carbon and ketone-containing functional groups and electrochemical oxidation on the CND surface. CNDs generally have a spherical shape, but varying synthetic methods can give crystalline structures [3,5,6]. These functional groups help polarize the CND, making it hydrophilic and thus readily dispersible across most biological membranes. CNDs have an inner sp^2^ and outer sp^3^ hybridized structure that often has oxygen-containing functional groups. Their surface imparts many qualities to CNDs, including easy electron transfer, which can give them anti- or pro-oxidant behavior [7]. The chemical and physical configuration of CNDs enables applications in fields from engineering to medicine. Their optical properties allow their use in bioimaging and biosensing, which has many uses in nanomedicine and pharmacology. CNDs have excitation-in/dependent photoluminescence that varies with structure, surface groups, defects, and environment. They have been used in the “theranostic” approach that involves sensing and delivery and have peroxidase-like activity [8]. Thus, a review of their synthetic methods and biologically relevant properties is essential for future use. This review is mainly focused on the developments of in the understanding of mechanisms of CNDs free radical scavenging and the role of functional tailoring of CNDs by modifying dopants and surface molecules in understanding of their antioxidant behavior and mechanism of action. We also discuss the emergence of research analyzing CNDs as useful therapeutic agents in various disease states.

## 2. Synthesis

**Figure 2:**
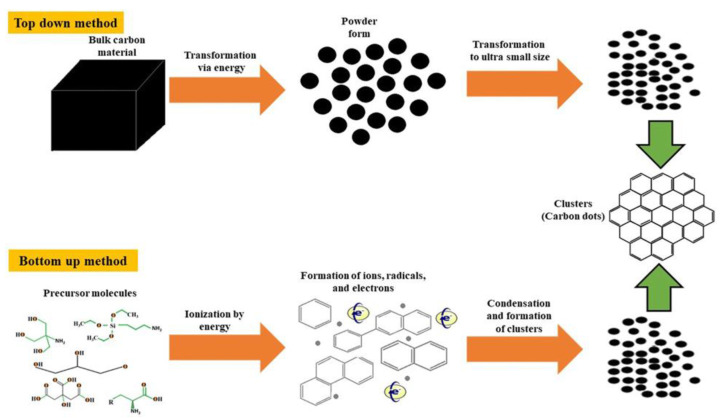
Top-down and bottom-up CNDs synthesis methods. Adapted with permission from Reference [19]. Copyright 2019 Springer Nature.

The first carbon dots (CNDs) were synthesized accidentally in 2004 by Xu et al. in an attempt to purify single-walled carbon nanotubes [9]. The experiment involved the use of arc-discharge soot as a source of carbon nanotubes. In the process, gel electrophoresis was utilized to separate the components of the soot suspension, revealing a novel band of fluorescent material. Current methods of synthesizing CNDs can involve chemical or physical processes. Chemical methods include thermal treatment, electrochemistry, acidic or hydrothermal oxidation, or ultrasonic treatment. Physical methods involve arc discharge, plasma treatment and laser ablation [10]. As shown in Figure 2 and Table 1, the methods of preparing CNDs can be classified broadly into top-down or bottom-up syntheses [11,12,13,14,15,16,17,18,19]. Top-down methods generally involve a larger carbon source being fragmented into increasingly smaller particles by using hydrothermal or solvothermal cutting, laser ablation, chemical oxidation releasing or etching, and intercalation. Bottom-up approaches involve carbonization of organic precursors to synthesize CNDs. These include ultrasonication, solvent-mediated microwave pyrolysis and refluxing pyrolysis, as well as dehydration with sulfuric acid. Organic precursors, such as natural gas, allotropic carbon forms, and carbohydrates, are converted into CNDs, using solvothermal or direct thermal decomposition [20]. The resulting product can undergo centrifugation, dialysis, chromatography, electrophoresis, and other methods to give uniformity [21].

CNDs are relatively easy to functionalize, which allows expansion of their therapeutic and biosensing capability [10]. The morphology and structure of CNDs is heavily dependent on the precursor, method of preparation, and experimental conditions. For example, CNDs produced from top-down methods have diameters and heights depending on the source material (graphite powder, graphene nanosheets, or coal). A singular preparation method such as etching can use UV irradiation or hydrothermal treatments, both of which would produce differential results. Generally, top-down-synthesized CNDs have sizes below 10 nm, have a spherical or sheet structure, and heights below 3 nm [20]. Bottom-up-synthesized CNDs are also sized less than 10 nm, and data suggest that these CNDs may be layered with heights of less than 10 nm [20]. Oxidation is a common factor in numerous synthetic procedures, resulting in CNDs exhibiting surface groups containing hydroxyl, carboxyl, and carbonyl groups. These groups give CNDs hydrophilic qualities, which expands their use in biological systems and aqueous solutions. Most research has been conducted on hydrophilic CNDs, but hydrophobic CNDs have been synthesized by Cayuela et al., using dodecyl-grafted poly (isobutylene-alt-maleic anhydride) (PMA) in 1-octadecene. The amphiphilic PMA polymer and one-step solvothermal reaction at 200 °C in organic solvent led to hydrophilic, fluorescent CNDs. The size of these nanoparticles increased with reaction time from 0.5 to 2 h, corresponding to a size of 3.4–4.3 nm [22]. Additional elements such as nitrogen, sulfur, and boron have been added to improve the capabilities of CNDs and increase quantum yield [7].

### 2.1. Top-Down Syntheses

As top-down methods, arc-discharge and laser ablation are arguably the most popular methods in the synthesis of carbon-based nanomaterials. Arc discharge denotes the action of producing a current between two electrodes (usually graphite rods), which leads to their vaporization. As a result, soot is produced, which may contain different carbon-based nanomaterials. Laser ablation releases nanomaterials by irradiating a solid surface with a pulse laser. Laser ablation in solution (LAS) garnered interest as a single-step top-down approach for the speedy and cheap generation of nanomaterials. Among its advantages, LAS is mostly contamination-free and reduces the formation of byproducts [23]. Gonçalves et al. performed laser ablation in a solution of water, N-acetyl-l-cysteine (NAC) and NH_2_-polyethylene glycol (PEG_200_) to produce passivated CNDs [20]. Nguyen et al. have analyzed changing parameters of this technique and their effects on carbon nanodots. For instance, decreasing laser fluence and spot size reduces the mean size of CNDs. Additionally, increasing irradiation time leads to a decrease in size, as well as the production of multiple functional groups in CNDs [23]. Precursors such as carbon-based cement and graphite powder have been widely used for arc-discharge, high-energy ion beam radiation, and laser ablation [24].

Multiple other approaches have been researched for top-down CND synthesis. Chemical oxidation involves the use of a strong acid to oxidize aggregates of carbon nanostructures, and as a result, introduce hydrophilic functional groups containing oxygen. The carbon nanostructures thereby become water-soluble, which facilitates their release into the solution. Xu et al. obtained soot from arc-discharged materials and oxidized it with 3.3 M HNO_3_. Following extraction with NaOH solution, the black suspension was purified by gel electrophoresis, revealing luminescent CNDs [20]. Electrochemical methods employed redox potentials high enough to oxidize C-C bonds or water to generate oxygen radicals. Ding et al. used multi-walled CNTs synthesized by chemical vapor deposition as electrodes to obtain CNDs. The economic and practical advantages of this method led to various developments in terms of carbon sources and electrode composition. Cycling potentials (–2.0 and +2.0 V) were applied to multi-walled CNTs on carbon paper in tetrabutylammonium perchlorate to prepare blue-fluorescent CNDs [20]. The size of CNDs was dependent on the current density from 20 to 180 mA cm^−2^, with larger CNDs emerging from low current values [5]. Hydrothermal and solvothermal cutting methods have also been explored. Pan et al. were among the first to employ these methods, using graphene sheets to produce CNDs by means of hydrothermal cutting at 200 °C for 10 h in 2010 [20]. One-step pyrolysis and hydrothermal treatment using leeks gave rise to blue and green-fluorescent CNDs, which resulted in allowed for ease in controlling reaction conditions and tunable PL [25]. Methods of intercalation are also among popular top-down approaches. These methods typically involve the placement of an element or compound within the sources of carbon, such as graphite sheets. Lin et al. prepared hydrophilic CNDs from CNTs and graphite flakes by using intercalation with potassium. Graphene sheets and intercalated K atoms made a compound that reacted with ethanol to disassemble the walls of CNTs. Ultra-sonication was used to further break down the walls and produce CNDs, among other byproducts. Other top-down methods express high levels of precision. Ponomarenko et al. cut graphene into specific sizes, using ultrahigh-resolution beam lithography. The direct synthesis of CNDs was achieved by Li et al. by hydrogen peroxide-mediated ultrasonic treatment of activated carbon [20]. Liu et al. used commercial graphic nanoparticles and vortexed them in ethanol and water, centrifuged the solution at 2000 RPM for 30 min, with a resulting supernatant of sp^2^ CNDs with the pure crystalline structure without defects [20].

### 2.2. Bottom up Syntheses

Bottom up methods has been thoroughly developed as an alternate economic system to produce CNDs from organic material. The starting materials and conditions generally result in CNDs below 10 nm that are photoluminescent and stable in an aqueous solution [20]. Gianneli et al. used hydrothermal heating of citric acid monohydrate (CA) and organic ammonium, which resulted in 7 nm CNDs after an acetone wash and filtration. After this initially reported synthesis of CNDs, the development of hydrophilic CNDs has grown to involve polyethylene glycol diamine (PEG), urea, ethanolamine, cysteine, glycine, ethylenediamine, and other organics [20].

Microwave pyrolysis was first used as a mode of synthesis by Zhu et al., using a dissolved saccharide and PEG-200. This solution was heated in a 500 W microwave oven, with the size of CNDs increasing with reaction time. Microwave pyrolysis reduces the reaction time, decreases side reactions, and increases the yield of CNDs [22]. However, some CNDs that were synthesized by using this method have blue emission and weak luminescence [26]. Bright emission CNDs were synthesized by Qu et al. by heating carbohydrates and inorganic ions for several minutes [20]. Similar methods involve heating organic molecules in solution at 300–900 W for an amount of time from seconds to minutes. Reactants have included poly (ethylene glycol) (PEG-200), carbohydrates with inorganic ions or 4,7,10-trioxa-1,13-tridecanediamine (TTDDA), amino acids and citric acid. Stefanakis et al. employed aqueous solutions of citric acid and urea or 6-aminocaproic acid and urea for a microwave heating process. Solutions were heated in a microwave oven to generate CNDs. The core of the CNDs is believed to have arisen from the citrate molecule. Other organic molecules comprised the surface groups to give a hydrophilic CND [27]. After the product is obtained, it is usually ultra-filtered, centrifuged, or dialyzed to produce purified CNDs. Surface passivation could be used to add polymers, generally using minutes of time [20]. Chen et al. microwave-synthesized CNDs from α-lipoic acid, citric acid, and urea resulted in N,S-co-doped CNDs with OH and NH surface groups that made them hydrophilic [8]. Microwave pyrolysis and laser ablation give CNDs that have strong graphite components and sp^2^ character as measured by XPS [27].

In fact, many variants of CNDs have been synthesized by using direct thermal decomposition where precursors are heated in an inert environment and carbonized. They are then extracted by using solvents, and TEM images show a general size of less than 10 nm, which is one of the reasons this method is extensively used [20]. In 2008, Gianneli et al. designed a method of direct thermal decomposition to synthesize CNDs, but they were irregularly shaped and only stable in organic solvents such as DMF and DMSO. Hydrophilic CNDs were then synthesized by using a different method of thermal decomposition in which citric acid was reacted with sodium 11-amminoundecanoate, heated and then centrifuged. This created core-shell structured CNDs of an overall size of 10–20 nm and core size of 5–10 nm [20]. Citric acid and tris(hydroxymethyl)aminomethane (Tris) were used as carbon and nitrogen sources for the one-step synthesis of nitrogen-doped CNDs (N-CNDs) [10]. Monodisperse spherical mesoporous silica particles (MSMSPs) were used to make CNDs by template synthesis. Organosilane was deposited into the pores and underwent thermal decompression, and subsequent template removal, which resulted in spherical CNDs sized 3.3 ± 0.9 nm [21].

The supported synthetic method used by Liu et al. involves the use of polymer/silica composites as carriers and phenols or formaldehyde resins as carbon precursors. Heating led to carbon/silica mixtures which then produced CNDs after etching with NaOH [20]. The following steps of oxidation, neutralization and purification were complex and time-consuming. This process was made simpler by Li et al., using nanoreactors of silica spheres having pores of 2–50 nm. They photoluminescent, stable, and relatively safe CNDs without any further purification reactions [20].

Concentrated H_2_SO_4_ has also been utilized to synthesize various CNDs, since it is a strong dehydrating solution. Zhang et al. first reported this method that involves using sucrose solution, adding sulfuric acid, neutralization, and dialysis. Pure CNDs were separated by molecular weight, which indicated their PL: 2 nm CNDs had green PL emission, and 5 nm CNDs were PL after passivation with PEG2000 N [20]. Other dehydration syntheses can involve ethylene glycol, ethylenediaminetetraacetic acid (EDTA), or bovine serum albumin as organics. Some also use heat, ranging from reaction temperatures 40 to 200 °C [20].

Many other methods have also been developed to synthesize CNDs of uniform size distribution and relatively high purity. Kim et al. etched graphene into CNDs by using thermal plasma jets and used ethylene gas and Ar plasma to make an atomic beam of carbon. Jiang et al. used commercial coffee, dissolved it in 90 °C water and centrifuged it at 14,000 RPM, and obtained ~4.4 nm CNDs after gel filtration chromatography [20]. Ultrasonic treatment was used by Li et al. to synthesize <5 nm CNDs from glucose. The acid/alkali assisted ultrasound generated pressure in solution, which can cause shearing forces to polymerize and carbonize glucose [20]. Crosslinked hollow CNDs were synthesized by Fang et al. by combining acetic acid, diphosphorus pentoxide, and water. Uniform CNDs were made by Lu et al. on a ruthenium surface, using C_60_ molecules, which reacted strongly enough to allow C_60_ molecules to embed and fragment into carbon clusters that eventually developed into CNDs [20].

**Table 1:** Examples of various precursors and synthesis methods of CNDs and their key applications.

Sources	Synthetic Approaches	Size (nm)	Applications	References
Citric acid and urea	Microwave synthesizer	2.4 ± 0.3 nm	Radical scavenging	[28]
Carbon fiber powder, HNO_3_	Hydrothermal	1.8 ± 0.3 nm	Bioimaging	[29]
Citric acid and glutathione	Hydrothermal	2.1–4.6 nm	ROS scavenging, anti-inflammation	[30]
Green chili (bell pepper, *Capsicum annuum*)	Microwave	2.6 ± 1.2 nm with irradiation timing from 2.5 to 3 min	Bioimaging, ROS scavenging	[31]
Chitosan and different unsaturated amides or carboxylic acids	Hydrothermal	2.6 ± 0.9 nm	Bioimaging, antioxidant	[32]
Glucosamine hydrochloride	Microwave synthesizer	2.0 ± 0.7 nm	ROS scavenging	[33]
Phenylenediamine	Hydrothermal	4.92 nm	Antioxidant, protection against kidney injury	[34]
Citric acid and ethylenediamine	Hydrothermal	1.9 ± 0.2 nm	Theranostic cancer applications	[35]
Citric acid and polyethyleneimine	Hydrothermal	9.8–9.9 nm	Bioimaging in vivo and Photothermal/photodynamic synergistic cancer therapy	[36]
Citric acid and polyethyleneimine	Microwave	~5 nm	Enhancement of the bioavailability of curcumin	[37]
Milk	Hydrothermal	~20 nm for CNDs-Doxorubicin complexes	Drug delivery	[38].
Exopolysaccharides	Hydrothermal	4.3 nm	Microbial viability assessment	[39]
Beer yeast power	Hydrothermal	less than 5 nm	Imaging of bacteria	[40]
1,4-naphthalenedicarboxylic acid and urea	Hydrothermal	3.5 nm	Assessment of microbial viability	[41]
Citric acid and urea	Microwave	2.4 nm	Antioxidation, ROS scavenging	[3]
Citric acid and ethyleneimine	Microwave	2.4 nm	Detection of Fe (III) ions	[42]

## 3. Photoluminescent (PL) Properties of CNDs

### 3.1. PL of CNDs

Nucleation and growth under different reaction conditions can result in CNDs with unique ratios of nitrogenous or oxygen-containing groups, which have their own photoluminescent (PL) qualities. The synthetic conditions of CNDs such as reaction time, temperature, and precursor materials all dictate their excitation wavelength, quantum yield, and photoluminescent (PL) [7,10,43,44,45,46]. The average absorbance of CNDs in the UV region is 330–420 nm. It is influenced by photon harvesting and has a tail in the visible region. However, π–π* bond and ketone group transition can cause CNDs to have a 250–300 nm band. Their emission wavelength is dependent on size and generally within 400–600 nm [24].

CNDs of varying size and synthetic methods have shown λ_em_ in the UV to NIR region, with Sun et al. demonstrating the PL intensity of CNDs relating to size. The size dependence of CNDs arises partly due to the transition gap between the lowest unoccupied molecular orbital (LUMO) and highest occupied molecular orbital (HOMO), which is inversely proportional to size [5]. CNDs with irregular size distribution have undergone gel electrophoresis and HPLC to determine the PL of 1.2 nm CNDs in the UV region and 1.5–3.8 nm CNDs in the visible and NIR regions [5]. Moreover, 30–50 nm CNDs had lower PL than 10 nm CNDs, which can be explained by the larger surface area to volume ratio in smaller CNDs, giving them higher PL [20]. Graphene sheets were hydrothermally cut to give CNDs with a size distribution of 5–17 nm and corresponding PL wavelength from 450 to 486 nm. This reversal of the quantum size effect was due to the shape change of the CND from circular to polygonal, causing an increase in PL energy [5]. Thus, the edge type of differentially prepared CNDs can influence PL even across varying sizes and shape. CNDs synthesized from flower petals had absorptions from 220 to 400 nm and strong green emission at 510 nm when λ_ex_ was 470 nm. This may be due to emissive defects and a high graphite content decreasing the band gap [47].

The λ_ex_ dependence of coffee-ground-synthesized CNDs was evident by their changing emission spectra from 400 to 600 nm [24]. Excitation dependence of CNDs may arise from the core and surface functional groups, as well as electronic state transitions on the surface [48]. This property distinguished them from other nanomaterials, such as SQDs, Au-nanodots, and nanoclusters [5]. Li et al. used graphene oxide reduced via hydrazine hydrate and functionalized the surface by using polyethylene glycol (PEG). When the modified CND absorbed multiple longer wavelength photons (600–800 nm) at once or sequentially, they emitted at a shorter wavelength (390–468 nm) [20]. Oil-synthesized CNDs and CU/CA-CNDs had tunable emissions that decreased fluorescence intensity with higher excitation due to weak absorption of CNDs in the visible region [27]. This indicated that long λ_ex_ could be used to penetrate deep tissues, minimize background noise, and be minimally toxic to be useful in vivo. Petal-synthesized CNDs showed a red shift and PL intensity increase and subsequent decrease at λ_ex_ from 430 to 490 nm. Moreover, the fluorescence lifetime (τ) of CNDs was 5.8934 ns on average [47]. Hydrophobic N-doped CNDs were excited from 250 to 600 nm and reached a maximum intensity at λ_ex_ of 390 nm and had a red-shifted emission [22]. They also showed upconversion, which is unique to CNDs and allows living cells to be excited at lower energies, decreasing the damaging effects and assisting in the removal of interference. The N-doped CNDs showed an emission peak at 500 nm, which then narrowed and showed a red shift when excitation wavelength changed from 700 to 900 nm.

### 3.2. Mechanism of PL

Differential synthetic methods give CNDs with unique sizes surface states, and this gives rise to unique photophysical qualities. The PL of CNDs has been theorized to emerge from special structure sites, surface states, conjugation, and many other sources. The combined effects of surface states, core composition, and surface groups are thought to comprise PL qualities of CNDs [10]. Some of the explanations for excitation-dependent fluorescence are π-domains with abundant π-electrons and energy gaps from bond disorders [48]. CND photoluminescence is widely understood to arise from a quantum confinement effect and emissive areas on the surface [24]. The surface state mechanism explains the PL due to surface electrons and holes in the surface of CNDs undergoing radiative recombination [20]. The surface hydroxyl and ether groups in most CNDs contribute energy gaps that lead to decreased excitation energies, causing red-shifted PL in the surface bands and blue PL in intrinsic bands [49]. The FL Sun et al. proposed that the passivation of CNDs with functional groups introduces defects in the surface that can become reservoirs of energy. These sites can produce photons due to radiative recombination, which produces PL after functionalization of the CND. Sun et al. induced low PL CNDs to emit bright PL by passivating the surface with organic polymers [20]. The resulting surface underwent radiative recombination, and this led to their PL. Lingam et al. were able to quench PL emission of CNDs by annealing the edges of CNDs in H_2_ at 250 °C. Thus, the surface of the CND being oxidized, functionalized, or chemically modified plays a key role in its PL properties.

The PL properties of a CND can be altered by its chemical or physical environment or the structure of the CND itself. The pH of the solution is one such factor that can cause PL intensity to increase with alkalinity. Some CNDs were strongly PL in neutral solutions, while others were independent of their solution’s pH and displayed a uniform PL [20]. Conversely, solvothermally produced Graphene oxide-CNDs had a constant PL in solutions of pH 4.0–8.0 and decreased PL otherwise [5]. The pH-dependent PL of CNDs can also be due to carbene-like zigzag sites that become protonated in acidic solution, resulting in the breakdown of their emissive state and quench the PL. CU/CA-CNDs was relatively stable from pH 2.2 to 11.5, with the highly acidic solution showing a decrease in absorption. This can be explained by deprotonation of amide and carboxylic on the surface since the intensity was recovered after pH reversal [27]. Similarly, N,S-CNDs showed pH independence from values of 5–9 and a decrease in fluorescence otherwise. Neither coffee ground- nor APTMS-prepared CNDs were dependent on pH (3.0–10.0) and showed a constant PL intensity [5]. CNDs from flower petals shifted with pH (3.0–10.0) but showed stability in response to other ions in the solution [47]. This reaction or a lack thereof may depend on the synthetic method and surface composition of CNDs.

Most of the synthetic routes discussed previously lead to CNDs containing functional groups with oxygen, such as carboxylic acid, carbonyl, ether, and epoxy groups. As the oxygen content of the surface increases, the maximum λ_em_ shifts to the red region. Treating the CND surface with sulfur and nitrogen has also been used as a means to adjust λ_em_ and quantum yield [5]. Additionally, oxidation of CNDs corresponds with greater λ_ex_ dependence (280–380 nm) due to surface defects that trap excitons which then undergo radiative recombination. It has also been theorized that CNDs with higher oxidation have abundant surface states that give PL with different energy that varies according to excitation.

Additional factors such as cationic presence could also influence the emission intensity of CNDs. The application 0.02 M of transition metal ions Ni(II) and Co(II) to CA-CNDs reduced fluorescence and shifted the emission maximum, possibly due to the binding of –COOH groups [27].

N,S-CNDs have linear excitation-dependent properties with a high QY of 73% and bright blue photoluminescence when λ_ex_ = 365 nm. Their emission red shifts from 426 to 516 when λ_ex_ increases from 360 to 470 nm. Due to their narrow size distribution, the proposed PL mechanism is surface state emission, as evidenced by concentration-dependent emission [50].

**Figure 3:**
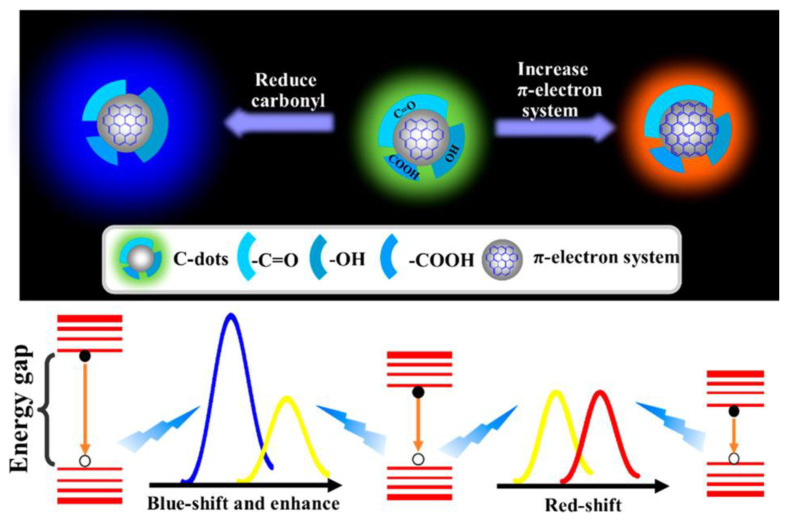
The PL mechanism, structure–performance relationship of CNDs. Adapted with permission from Reference [29]. Copyright 2019 ACS.

Control of surface functional carbonyl groups also significantly affect the PL characteristics of CNDs. Previous studies have shown that with the content of carbonyl group decreases, the PL of CNDs undergoes a blue shift, indicating that the carbonyl group can change the electron transition to n → π* type resulting its energy lower than that of π → π* type [29]. When the content of C=C increases, the degree of delocalization of the π-electron system can also be increased resulting in a decrease of energy gap and further leading to a red shift of the emission wavelength of CNDs [29]. Therefore, the carbonyl group and the π-electron system can be coupled with each other (Figure 3), changing the electronic state of the CNDs and further affecting the emission wavelength [29].

## 4. Antioxidant Properties and the Underlying Mechanisms

Among the key characteristics that permit CNDs a variety of biomedical applications, their antioxidant properties to protect cells from oxidative stress are a curious one. Recent studies have characterized CNDs’ ability to scavenge a variety of ROS radicals, including superoxide and hydroxyl anions. Even so, there is much yet to be understood about the mechanism of action, which would further their use in biomedicine.

### 4.1. Antioxidant Properties of CNDs Scavenging Free Radicals

Reactive oxygen species (ROS) are radicals, as in the case of the hydroxyl radical (OH) or superoxide (·O^2−^). Others can be oxidizers, such as hydrogen peroxide (H_2_O_2_) and peroxynitrite (ONOO^−^) [51,52]. ROS are essential components of cells and help to signal pathways throughout the body. However, overproduction of ROS disrupts homeostasis, has been shown to be one of the key contributing factors to its progression of many chronic and degenerative diseases process such as cardiovascular diseases, cancer, neurodegenerative, and diabetes.

**Figure 4:**
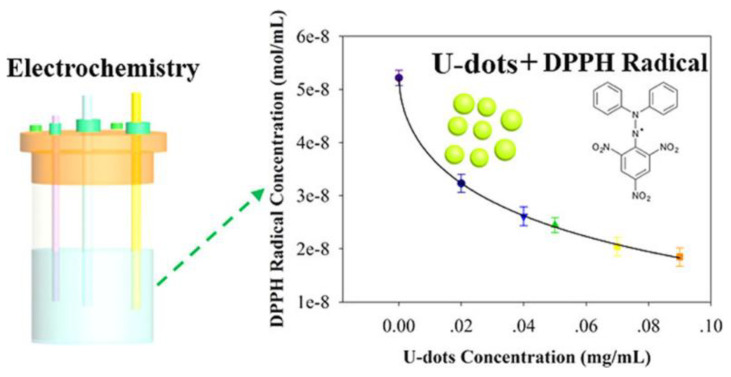
DPPH radical scavenging by CNDs. Adapted with permission from Reference [28]. Copyright 2017 ACS.

Although nanomaterials can be sources of excessive ROS production, some CNDs have shown antioxidant activity in biological systems [3,26,30,31,53]. The conjugated π-system allows CNDs to behave as electron donors, which enables free-radical scavenging or antioxidant behavior [54]. The antioxidant properties of CNDs are dependent on their behavior as electron donors or acceptors. Zhang et al., performed an electrochemical study investigating interactions between DPPH· and CNDs [28]. CNDs were synthesized from microwave pyrolysis of urea and citric acid via a one-step microwave-assisted process using citric acid and urea. Characterization followed using transmission electron microscopy (TEM), determining an average size of ~2.4 nm. FTIR spectra determined the presence of functionalities, including -COOH and -NH_3_, which are indicative of stability and hydrophilicity in aqueous media. Antioxidant activity was inspected via UV–Vis spectroscopy. The absorbance of DPPH· was observed to decrease with increasing concentrations of CNDs (002 to 0.09 mg/mL), indicating a dose-dependent correlation that plateaus at higher concentrations [28]. These results demonstrated dose-dependent scavenging of 2,2-diphenyl-1-picrylhydrazyl radicals (DPPH·) by CNDs (Figure 4).

**Figure 5:**
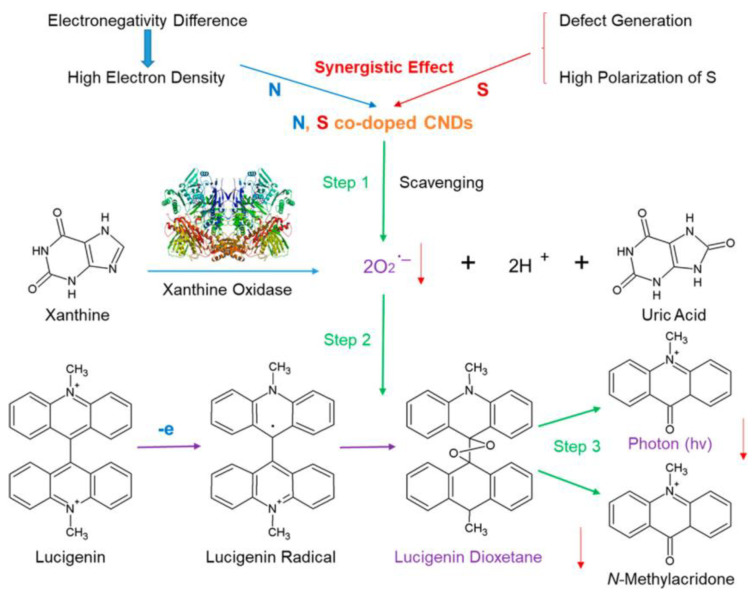
Schematic diagram of the reaction pathway of CNDs scavenging superoxide in the xanthine/xanthine oxide (XO) system. Adapted with permission from Reference [6]. Copyright 2018 ACS.

Zhang’s group also analyzed the scavenging ability of superoxide anions by using lucigenin-CL assays (reaction of xanthine with xanthine oxidase enzyme produces O_2_−). The detection of ROS by using the Lucigenin-CL assay showed a dose-dependent decrease in CL intensity from 0.05 to 0.10 mg/mL, which indicates their superoxide scavenging activity [6]. Increasing concentrations of N,S-CNDs showed lucigenin-CL quenching, with 59% decrease at 0.10 mg/mL CNDs. Though efficient, the antioxidant capacity of DPPH· was notably higher (~90% vs. ~59%). Zhang’s group proposed that reducing lucigenin upon interaction with the CNDs creates a lucigenin cation radical that can then react with XO-derived superoxide radicals (Figure 5).

CNDs scavenged oxide radicals in a dose-dependent manner as measured by a KMnO_4_ degradation assay. The DPPH∙ assay using a BHT standard showed that CNDs have comparable antioxidant activity at a much lower concentration than the standard [26]. The DCFH-DA assay determined that CND inhibition of hydroxyl radicals (OH) was better than the L-ascorbic acid standard and increased logarithmically. The NBT assay showed superoxide scavenging by CNDs also followed a dose-dependent logarithmic increase with an EC_50_ value of 1.75 mg/mL. Thus, the NBT assay can be used to measure oxidative stress in vitro by using CNDs as optical probes. CNDs from chitosan fed to zebrafish (200 µg/mL) resulted in a 40–60% reduction in SOD activity and 120% reduction in ROS activity. The catalase-like activity of the CNDs from using electron paramagnetic resonance (EPR) showed a dose-dependent (7.6–15.2 µg/mL) reduction in the production of OH radicals [55].

### 4.2. Antioxidant Properties of CNDs Protect against ROS-Induced Cellular Oxidative Damage

**Figure 6:**
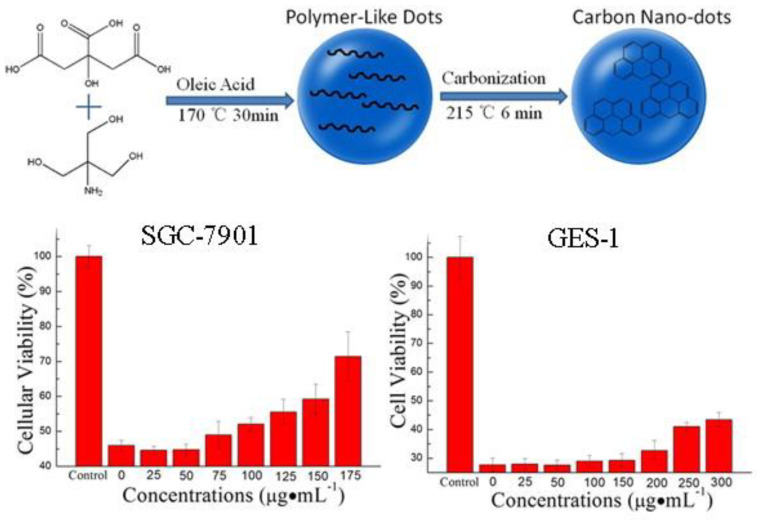
CNDs protect against H_2_O_2_ toxicity in SGC-7901 and GES-1 cells. Adapted with permission from Reference [10]. Copyright 2015 ACS.

Several studies have reported the antioxidant properties of CNDs protect against ROS-induced oxidative damage [10,30,56]. For example, the cytoprotective effect of N-CNDs was examined by the MTT assay in SGC-7901 and GES-1 cells treated first treated with 2 mM H_2_O_2_ (Figure 6). The addition of N-CNDs significantly increased cell viability from 28% to 43% in a dose-dependent manner [10].

**Figure 7:**
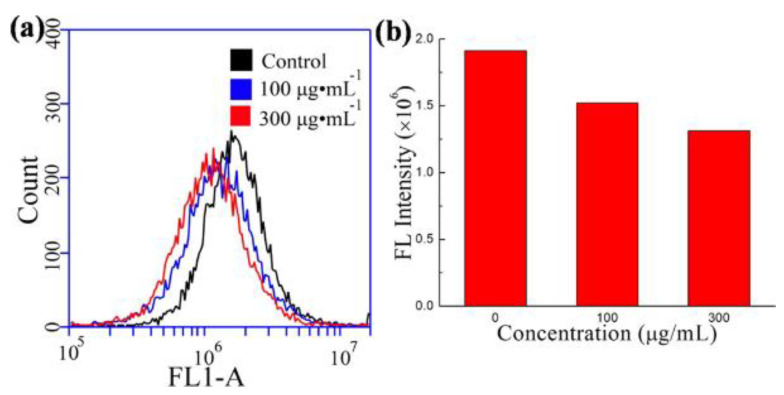
CNDs decreased ROS in SGC-7901 cells: (**a**) ROS detection by flow cytometry and (**b**) ROS detection by fluorometric assay. Adapted with permission from Reference [10]. Copyright 2015 ACS.

These cancer cell lines can have greater metabolic rates and thus ROS production, and the quenching of CNDs and stimulation of antioxidant defense systems can be helpful in treating oxidative damage. This was demonstrated by the DCFH-DA assay, which allows fluorescent ROS to be detected intracellularly (Figure 7). Results showed ROS fluorescence decreased and SOD production increased with the addition of CNDs (0–100 µg/mL) [10]. CNDs from microwave irradiation of date molasses scavenged hydroxyl radicals and superoxide anions, which implies their use in cytoprotection due to oxidative stress.

### 4.3. The Mechanisms of CNDs Free Radical Scavenging

#### 4.3.1. H-Atom Transfer (HAT) Mechanism

**Figure 8:**
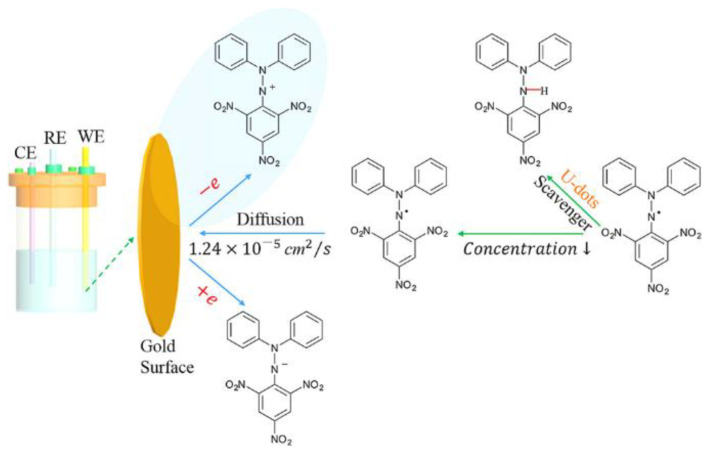
The electrochemical mechanism of CNDs in the scavenging free radical DPPH∙. Adapted with permission from Reference [28]. Copyright 2017 ACS.

CNDs have been designated as useful antioxidants in biomedical applications, but mechanisms of interaction still require much research. This study provided critical information that may further enable the development of CNDs as useful biomedical tools. To study the underlying mechanism of CNDs ROS scavenging, the electrochemical study performed by Zhang’s group consisted of incubating a DPPH methanolic solution with varying concentrations of U-CNDs and scanning for redox peaks in CVs [28]. With increasing U-CND concentrations, the anodic and cathodic peaks were observed to decrease in strength. Subsequently, the DPPH· concentrations were calculated as 32.3 to 18.5 nmol/mL with U-CND concentrations of 0.02 to 0.09 mg/mL, demonstrating increasing dose-dependent scavenging ability [28]. Additionally, each scan rate proposes that heterogeneous electron transfers are taking place. Zhang’s data exclaim that, in the presence of U-CNDs, DPPH is converted into DPPH-H by way of a HAT mechanism (Figure 8). This is facilitated by active surface groups that act as proton donors. Conclusively, these results support previous UV–Vis spectroscopy experiments and provided novel evidence aiding in the understanding of the antioxidant behavior of CNDs [28].

Doping CNDs with nitrogen and sulfur: N-doped CNDs embedded in ionic solution also scavenged radicals due to their amino surface groups. Doping CNDs with nitrogen and sulfur improves their scavenging ability by increasing their ability to donate electrons [6]. The antioxidation behavior of N,S-co-doped CNDs was examined by using a variety of assays [6]. A hydrothermal method was used to prepare the CNDs, using α-lipoid acid, urea, and citric acid. FTIR spectra demonstrated broad bands, indicating N-H and O-H functionalities, which contribute to increased hydrophilicity. Additionally, the CNDs exhibited a spherical morphology doped with N and S functionalities. In order to evaluate the antioxidation activity of these CNDs, a UV–Vis spectroscopy of DPPH· was utilized. Mixing DPPH· with N,S-CNDs in a methanol solution resulted in a decrease in the absorbance intensity of DPPH·. Antioxidant activity was boosted with increased concentrations of CNDs. Zhang’s group suggested that due to the stronger electronegativity of nitrogen, higher positive density is placed on carbon atoms, which enables interaction between N-doped CNDs and DPPH· [6]. The addition of sulfur also increases the polarizability of CNDs, further expediting their interactions. The synergistic effect of N and S doping is thought to promote the π system to function as an electron donor.

N-doped CNDs increase in synthesis yield and inhibit ROS: Previous studies demonstrated the protective effect of N-doped CND on zebrafish under oxidative stress via downregulating intracellular ROS (decrease by 68%), malondialdehyde (MDA), and stimulating the production/expression of intracellular antioxidant enzyme catalase [32]. MDA is a widely used as lipid peroxidation oxidative biomarker. Catalase is known to decompose hydrogen peroxide into water and oxygen. Excessive hydrogen peroxide can significantly reduce the protective effect of catalase, which is related to the development of a series of health conditions, including cancer, diabetes, cardiovascular diseases, and neurodegenerative diseases such as Alzheimer’s disease and Parkinson’s disease. Doping of nitrogen elements into the CNDs is in the form of pyridine-like N (74%) and NH2 (26%) [32]. Carbon–carbon double bond significantly increases the synthesis yield of N-doped CNDs reaching to 85.9% [32]. With the increase of C=C content from 14 to 56 mmol the synthesis yield increased by 3.3 times. The conjugated π-systems suitable for electron donors and its pyridinic-like structures are both potential mechanisms of CNDs to inhibit ROS [32].

#### 4.3.2. Role of COOH and -NH_2_ Functional Groups

Studies have proposed that the antioxidant activity of CNDs is primarily due to the properties of certain functional groups, such as primary amines and carboxyl groups. However, direct evidence denoting the involvement of these functional groups is lacking. Ji’s group altered these surface functional groups in CNDs to analyze their involvement in DPPH· reactions [33]. Ji et al. used glucosamine hydrochloride as a precursor and synthesized fluorescent CNDs with a green microwave process. Size distribution of unmodified CNDs was observed to be ~2 nm. Moreover, -COOH functional groups were blocked by reacting unmodified CNDs with 0.5 mM 1-(3-dimethylaminopropyl)-3-ethylcarbodiimide hydrochloride (EDC, TCI)/0.5 mM N-hydroxysuccinimide (NHS, Aldrich), and -NH_2_ groups were blocked by mixing with citraconic anhydride with ensuing pH adjustment. Moreover, -COOH-blocked and -NH_2_-blocked CNDs exhibited an average size of around 3 and 4 nm. Characterization studies of the blocked CNDs followed. In -COOH-blocked, CNDs, the O atom percentage increased from 5.63% to 5.74%, and the N atom percentage increased from 1.36% to 1.83%. FTIR spectroscopy was utilized to characterize similarities and differences across all three CND types. For instance, all types showed O-H and N-H functionalities, as well as C=C and C=O vibrations. Nonetheless, vibrations attributed to carboxyls and simple ketones shifted to imides in -COOH blocked CNDs. New peaks were also detected, indicating the presence of esters. Moreover, -NH_2_-blocked CNDs showed a reduction in C=C and C=O vibrations typically attributed to amides [33]. These and other results demonstrated blocking functional groups in the surface of the CNDs was successful.

The antioxidant potential of all CND types was analyzed via UV–Vis spectroscopy, using DPPH· reduction assay. Using CND concentrations of 0.02, 0.04, 0.06, and 0.08 mg/mL, it was observed that increasing concentrations of unmodified CNDs exhibited higher percentages of scavenging (24% to 66%. In contrast, -COOH-blocked and -NH_2_-blocked CNDs showed decreased antioxidant activity percentages with increasing concentrations (45% to 27% and 58% to 38%). Using cyclic voltammograms (CV), Ji et al. performed an electrochemical study of DPPH· scavenging. With increasing concentrations of unmodified CNDs, the observed peaks showed dramatic decreases. This was not so readily observed with the blocked CNDs. Using mathematical equations (Randles–Ševčík), the scavenging activity for different concentrations of each CND type was determined. At a concentration of 0.04 mg/mL, for instance, unmodified, -COOH-blocked, and -NH_2_-blocked CNDs performed with 21%, 9%, and 11% scavenging ability of DPPH·.

The results of this study cannot ascertain which of the two functional groups has more impact in antioxidant capability. In the UV–Vis assay, NH_2_-blocked CNDs expressed less antioxidant activity. The electrochemical assay, however, indicated the opposite. Regardless, the study does signify that both functional groups play a key role in the antioxidant capability of CNDs. Hydrogen atom transfer (HAT) mechanisms have been proposed as potential pathways for radical scavenging in CNDs. Here, Ji’s group proposed an indirect HAT mechanism, denoted by a single electron transfer that precedes proton transfer. From this proposition rose the notion that carboxyl and amino groups contribute to antioxidation through HAT mechanisms. This led to the prediction that protonation of said functional groups would increase DPPH· scavenging. Ji’s group tested this by performing the UV–Vis assay once again with all three CND types in different pH values (3,7 and 11). All three types showed a tendency of increased antioxidant activity in more acidic solutions. This was explained to be due to the increased protonation of carboxyl and amine groups in acidic solutions, providing more hydrogen atoms for antioxidation activity [33]. Thus, the N and S doping of CNDs (due to an increase in the π-system electron density) have an important effect on their fluorescence intensity and their free radical scavenging ability [6,28]. Accordingly, the results of this study provide a more concrete foundation in the understanding of antioxidant behavior in CNDs.

## 5. Therapy

Due to the growing potential of CNDs in biomedical applications, studies have attempted to discern their use as therapeutic agents [30,56,57,58,59,60,61].

### 5.1. Treatment of Acute Kidney Injury (AKI)

The application of CNDs has also seen its first potential as a therapeutic practice for the treatment of acute kidney injury (AKI). AKI is characterized by rapid kidney damage or failure within a matter of hours to days. AKI often sees a loss of excretory function and is commonly seen in hospitalized patients. This condition is associated with high mortality rates, and although reversible, recovering patients are often left dialysis-dependent or with severe renal impairment [62]. Besides renal dialysis and full kidney transplantations, no effective pharmacological therapy exists. With the population incidence only rising worldwide, AKI represents a growing health concern.

Renal damage in AKI is initiated by irregular and divergent generations of ROS. It is well-known that ROS misbalancing leads to cell apoptosis or necrosis. Its crucial role in the onset of AKI has made ROS a popular target for AKI treatment. Unfortunately, several tested antioxidants display limited efficacy due to kidney targeting and have been known to worsen renal trauma. Nanoparticle use has been attempted as a potential treatment of AKI. However, finding NPs capable of passing through both the glomerular endothelial fenestrae and podocyte slit diaphragm (~10 nm pores) remains challenging. Due to their less than 10 nm size, biocompatibility, and antioxidant ability, Gao et al. led an investigation into CNDs as a potential in vivo therapeutic agent to AKI [34].

First, Gao et al. synthesized four antioxidant CND types by using different precursors: PDA, (2,4-DAT), (2,6-DAT), and MPDSA [34]. Upon determining mean size, structural and chemical characterizations, photoluminescence, and cytotoxic and antioxidant properties, PDA-CNDs (*m*-phenylenediamine) were chosen for further research. Next, it was determined that PDA-CNDs entered HK2 cells and localized mainly in the cytoplasm. Simulating AKI in vitro with a sequential hypoxia–normoxia model, it was determined that HK2 cells allowed strong uptake of PDA-CNDs while maintaining acceptable cell viability at a high concentration [34]. The protective effects of PDA-CNDs were also evaluated in the hypoxia–normoxia model, as well as in an H_2_O_2_ model. Cell viabilities were increased by over 20% in both models, as well as a significant reduction in ROS levels in the H_2_O_2_ model [34].

The biodistribution of PDA-CNDs in healthy mice and IR (ischemia-reperfusion) AKI mice was evaluated in vivo. Upon inducing AKI in mice by unilateral renal pedicle clamping, both the control and experimental model received an intravenous injection of PDA-CNDs. Both models demonstrated accumulation mainly in the liver and kidneys. However, IR-AKI mice showed stronger uptake in their unilateral injured kidney as opposed to their uninjured contralateral kidney, as well as the unilateral kidney of uninjured mice. This analysis was followed by morphological observations of the renal cortex. PDA-CND injection into IR-injured kidneys showed reductions in cast formation, tubular necrosis, and dilation, and brush border loss. Upon surgical removal, IR-injured kidneys injected with PDA-CNDs also demonstrated improved renal function. This was determined by measuring blood-urea nitrogen and serum creatinine levels. ROS levels were also measured and found to be reduced in treated IR-injured kidneys [34].

**Figure 9:**
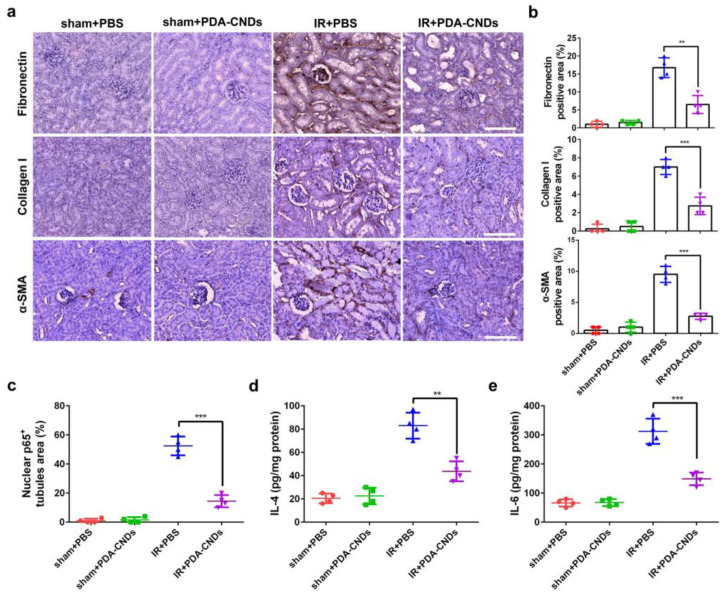
CNDs reduced inflammatory markers in renal tissues: (**a**) representative immunohistochemistry staining, Scale bar: 100 μm; (**b**) quantification of immunohistochemistry staining; (**c**) levels of nuclear NF-κB p65 in tubules area; (**d**) IL-4 levels in kidney tissues; and (**e**) levels of IL-6 in kidney tissues. (** *p* < 0.01, *** *p* < 0.001). Adapted with permission from Reference [34]. Copyright 2020 ACS.

To further the analysis of the therapeutic efficacy of PDA-CNDs on IR-AKI mice, inflammatory biomarkers were analyzed in paraffin-embedded kidney sections (Figure 9). Fibronectin, collagen I, and α-smooth muscle actin are denoted as markers for mesenchymal fibrosis. All three showed reductions in IR-injured kidneys with PDA-CND treatment. The same was observed for pro-inflammatory factors p-65, IL-4, and IL-6. Similar results were observed with a cisplatin-induced AKI mice model with treatment of PDA-CNDs. Lastly, the in vivo toxicity of mice was assessed with or without PDA-CND treatment. No apparent organ damage was observed in healthy, or PDA-CND treated mice at 24-h and 1-month time points [34].

**Figure 10:**
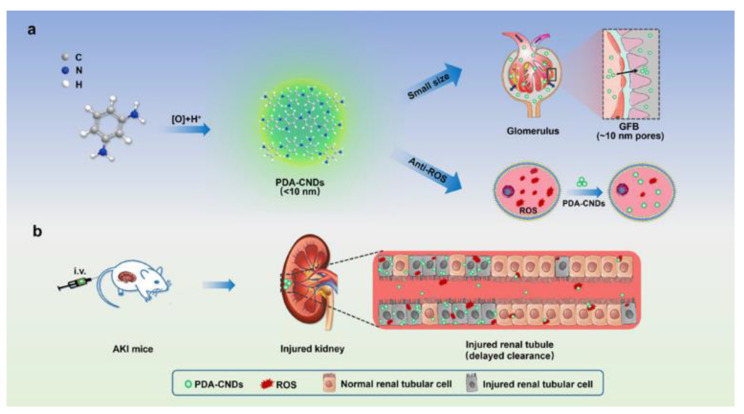
CNDs accumulate into kidney tissue and reduce acute kidney injury via antioxidant capacity: (**a**) antioxidative properties of CNDs in vitro and (**b**) therapeutic effects of CNDs against acute kidney injury. Adapted with permission from Reference [34]. Copyright 2020 ACS.

Collectively, this study denotes a novel technique in the pharmacological treatment of AKI. PDA-CNDs were chosen for this study due to their balanced ROS scavenging ability with low cellular cytotoxicity (Figure 10). Strong therapeutic efficacy was observed in two AKI models (IR-injury model and cisplatin-induced model). Importantly as well, the results of this study give promise to the notion that specifically designed CNDs can be potential therapeutic agents in different diseases and conditions.

### 5.2. Photothermal Therapy

CNDs have also demonstrated potential in photothermal therapy [35,36,63,64,65,66,67,68]. This process usually involves using a NIR laser to illuminate a tumor. The energy is then converted to heat through optical absorption. In time, due to the excessive heat, the tumor becomes partially or totally ablated [69]. Bao et al. utilized groups of mice with or without H22 tumors. One group was injected intravenously with N,S-co-doped CNDs and irradiated with a NIR laser, while another was not injected with CNDs but irradiated with a laser regardless. Upon analysis via thermal mapping, results indicated that the temperature in tumors of CND-injected mice was significantly higher than in mice not injected with CNDs (59–71 °C vs. 44 °C). This suggested that CNDs are involved in heat generation [70]. Perhaps most significantly is the analysis of tumor growth that ensued. Bao et al. then determined that after 8 days, tumors had vanished in mice treated with CNDs and irradiated with NIR laser in comparison with control groups. Treated mice also survived over 3 months in comparison to controls lasting 14–18 days. No major signs of inflammation or organ damage were also noticed in treated mice [70]. Collectively, this study provides ample indication for the potential of CNDs as anticancer tools, at least in the practice of photothermal therapy.

### 5.3. CNDs Based Nano Systems for Drug Delivery and Its Use in Cancer Theragnostic

Currently, most anticancer drugs are not specific to cancer cells to a large extent. In addition, these drugs often have huge adverse side effects on patients. In recent years, CNDs have attracted much attention because of their attractive properties, such as biocompatibility and low toxicity and their abilities to load a variety of drugs for drug delivery and cancer targeting. Curcumin(1,7-bis(4-hydroxy-3-methoxyphenyl)-1,6-heptadiene-3,5-dione), also known as diferuloylmethane, is a natural compound found in the rhizome of *Curcuma longa* (turmeric) and some plants in the Araceae family. A number of studies have shown that curcumin has anti-cancer properties in vivo and in vitro [71,72,73,74]. These studies indicate that curcumin can inhibit cell proliferation and induce cell cycle arrest and apoptosis through a variety of mechanisms [72,73,74]. However, a major challenge in using curcumin to treat cancer is its bioavailability [71]. Arvapalli et al. reported the use of carbon nanodots to enhance the bioavailability, release kinetics, and anticancer activity of curcumin [37]. Ethylene diamine-derived CNDs and urea-derived CNDs were synthesized and conjugated with curcumin. The adsorption efficiency and loading capacity of curcumin in percentage were the determined. The Korsmeyer and Peppas equations were used to study the release kinetics of curcumin from a curcumin–CNDs complex. Kinetic studies shown a pH-dependent release of curcumin over time [37]. The intracellular and anticancer activity was examined in cancer cell lines, HepG2 and A549 cells, and an endothelial EA.hy926 cells. Compared with natural curcumin, curcumin loaded with CNDs increases the bioavailability in the cells [37]. These results indicate that CNDs may be an important curcumin carrier, which can improve the bioavailability of curcumin and enhance its anticancer properties at low concentrations.

**Figure 11:**
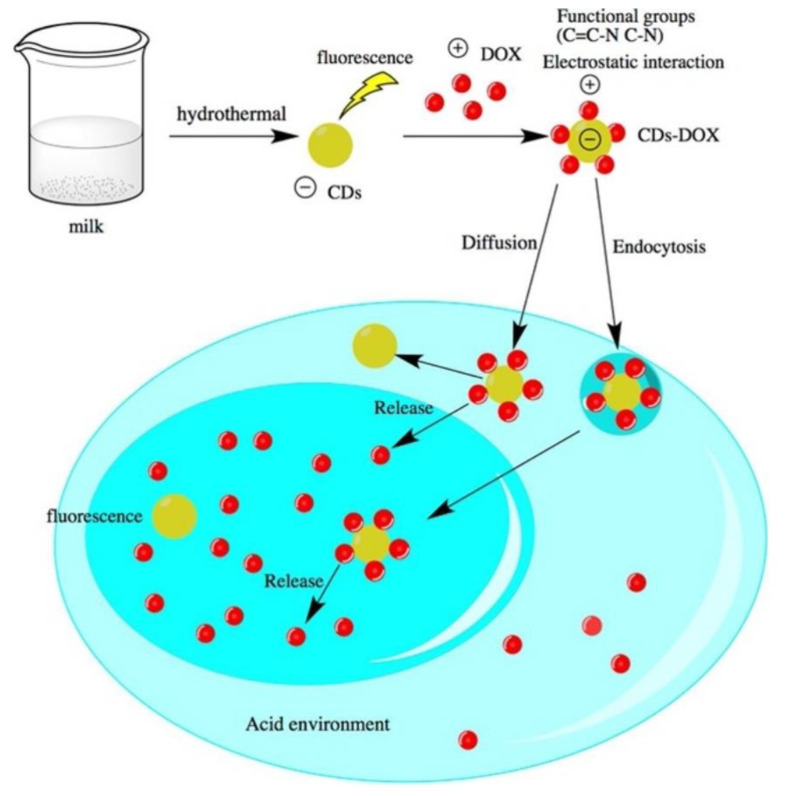
Doxorubicin-loaded carbon dots as a drug delivery system for targeted cancer therapy. Adapted with permission from Reference [38]. Copyright 2017 Elsevier.

Doxorubicin is an important antineoplastic agent due to its high effectiveness, but this effectiveness is overshadowed by its dangerous side effects. Yuan et al. used milk hydrothermolysis to synthesize CNDs and conjugated with doxorubicin via electrostatic interaction [38] (Figure 11). TEM, AFM, PL, FTIR, XPS, and XRD analysis confirmed the difference in physical and chemical properties between CNDs and CNDs–doxorubicin complex. The CNDs–doxorubicin complex showed a slow release of doxorubicin, which was dependent on pH [38]. Compared with free doxorubicin, it was found that the CNDs–doxorubicin complex can enhance the antitumor behavior of ACC-2 cells and reduce the cytotoxicity of L929 cells that are not sensitive to doxorubicin. Confocal scanning microscopy confirmed the focal accumulation of the CNDs–doxorubicin complex in the nucleus. In addition, the results of flow cytometry further support the enhanced anticancer ability of CNDs–doxorubicin [38].

## 6. Optical Imaging in Microbial Viability

CNDs also exhibit promising potential for optical imaging in microbial viability assessments [39,40,75]. Detection of microbial infections in modern medicine is performed by several techniques, such as atomic force microscopy, surface-enhanced Roman scattering, and PCR. However, these methods are denoted by complicated procedures, high expenses, and are time-consuming. Fluorescent dyes are typically used in microbial viability assessments due to their simplicity and convenience. Unfortunately, these dyes are often toxic, expensive, and unstable. Qie et al. have recently prepared nitrogen/phosphorus-co-doped CNDs (N,P-CNDs) as an efficient replacement of fluorescent dyes [41].

To begin, these CNDs were prepared by a one-step solvothermal method that uses 1,4-naphthalenedicarboxylic acid and urea dissolved in H_3_PO_4_. Characterization revealed these CNDs have a narrow size distribution (3–6 nm), high N and P doping, and strong blue fluorescence. The distinct functional groups give rise to different energy levels and surface states which give emission properties to these CNDs. Cytotoxic effects were determined via MTT assay of different CND concentrations up to 350 µg/mL. Cell viability remained above 85% at all concentrations. Photostability tests revealed little change to fluorescence intensity at increasing temperatures, UV irradiation time, and varying pH. Furthermore, a high QY marked the N,P-CNDs as promising bioimaging probes.

**Figure 12:**
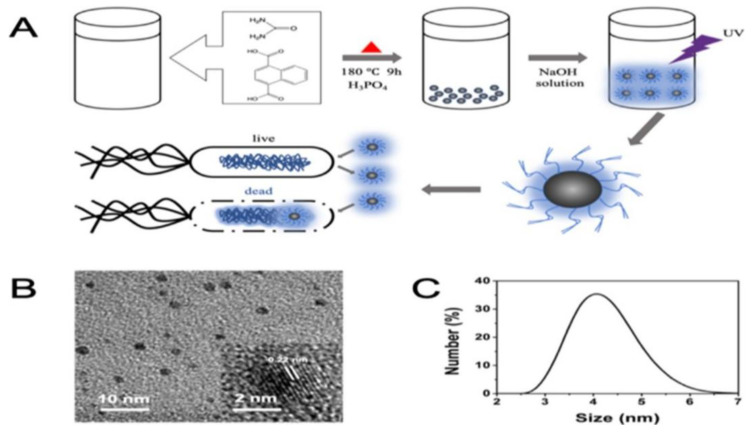
CNDs for detection of microbial viability: (**A**) diagram of CNDs synthesis and selective staining of dead bacteria by CNDs; (**B**) TEM image of CNDs; and (**C**) CNDs dynamic light-scattering spectra. Adapted with permission from Reference [41]. Copyright 2020 Elsevier.

Six types of bacteria cells were prepared in order to assess the performance of N,P-CNDs in the assessment of microbial viability (three G- and three G+), as well as two fungi types. Scanning with a confocal laser demonstrated fluorescence at different excitation wavelengths in dead bacteria cells. Living bacteria cells showed no fluorescence, successfully concluding N,P-CND differentiation of live and dead bacteria cells. Similar results were observed between live and dead fungi cells. Comparison with propidium iodide (PI), a fluorescent dye, followed. The N,P-CNDs and PI were applied to a mix of dead and live microorganism cells. Fluorescence of both materials was only detected in dead cells, leading Qie et al. to speculate that N,P-CNDs penetrate damaged cell membranes in a similar fashion as PI [41]. Overall, these findings suggest great potential for N,P-CNDs as a more efficient replacement of fluorescent dyes in microorganism detection (Figure 12).

## 7. Bioimaging

The low cytotoxicity of CNDs as compared to SQDs allows their use in bioimaging in vitro and vivo, which opens many possibilities in nanomedicine [3,76,77,78,79,80,81,82,83,84,85,86,87]. With their high quantum yield and excitation-dependent emission, CNDs can be used for multicolor imaging in specific cells or organs. Furthermore, CNDs co-doped with sulfur and nitrogen had fluorescence that was not influenced by changes in their chemical and biological environments. The surface groups on CNDs dictate their passage through biological membranes and thus overall distribution. CNDs synthesized by using folic acid and urea were able to target cells that had an abundance of folate receptors, which marked them as cancerous [7]. When CNDs were functionalized to enter the nucleus by using a signal peptide, results showed strong nuclear fluorescence in MCF7 and A549 cells.

### 7.1. In Vitro Bioimaging

Green synthetic CNDs from coffee grounds were tested in LLC-PK1 cells to determine their internalization. Confocal images revealed human uterine cervical cells localized CNDs in the cytoplasm and cell membrane, possibly through endocytosis [24]. This demonstrated the ability of CNDs to remain stable and emit strong PL in culture media. Then 16HBE cells were treated with oil-based CNDs, and fluorescence imaging showed cytoplasmic localization after excitation at 488 nm. However, the nuclear region was devoid of any fluorescence, signaling that these CNDs may not interact with or disrupt genetic material [8]. HeLa cells cultured with CNDs for 2 h underwent multiphoton excitation fluorescence microscopy (MPEF) and showed bright PL from the cytoplasm and cell membrane [27]. PPEI-EI-treated CNDs were incubated for 2 h in human breast cancer (MCF-7) cells and showed strong PL at 800 nm and cytoplasmic localization. Glycine–CNDs were also internalized by these cells at displayed varying colors of PL, according to λ_ex_, from 360 to 480 nm [5]. The fluorescent properties of molasses-produced CNDs were used for bioimaging in vitro and showed cytoplasmic distribution. They also showed hemocompatibility and could be used to image blood vessels and surrounding tissue structure [26]. Possible mechanisms of uptake can be endocytosis, but various factors pertaining to CND size and surface passivation can influence their passage through the plasma membrane. CNDs from plant petals with –COOH groups and the negative charge did not aggregate due to repulsion. They were tested in A193 cells (0.6 mg/mL) for 0.5 h and showed uniform green fluorescence with a λ_ex_ of 488 nm. The PL intensity was found in the cytoplasm and nucleus, which suggests that these molecules can readily cross membranes and interact with genetic material [47]. The cellular internalization of ROS-probing CNDs was indicated by strong PL after a 4-h incubation in HeLa cells, which demonstrates their permeability. Further imaging revealed the CNDs were localized in lysosomes, possibly after undergoing endocytosis by cells [49]. U-CNDs applied to HepG2 cells showed uptake by cells in 24 h, as monitored by spinning-disk confocal microscopy [48]. Red fluorescent CNDs were incubated for 6 h in NIH/3T3 cells and analyzed by fluorescence microscopy, which showed good potential for live bioimaging without interference or photobleaching [88]. Chitosan-derived CNDs showed dominant cytoplasmic distribution in cells, with weak fluorescence in other areas such as the nucleus [54]. OPD-CNDs were photostable and showed pH-sensitive PL intensity when applied to multiple cell lines, and fluorescence imaging showed internalized CNDs [89].

**Figure 13:**
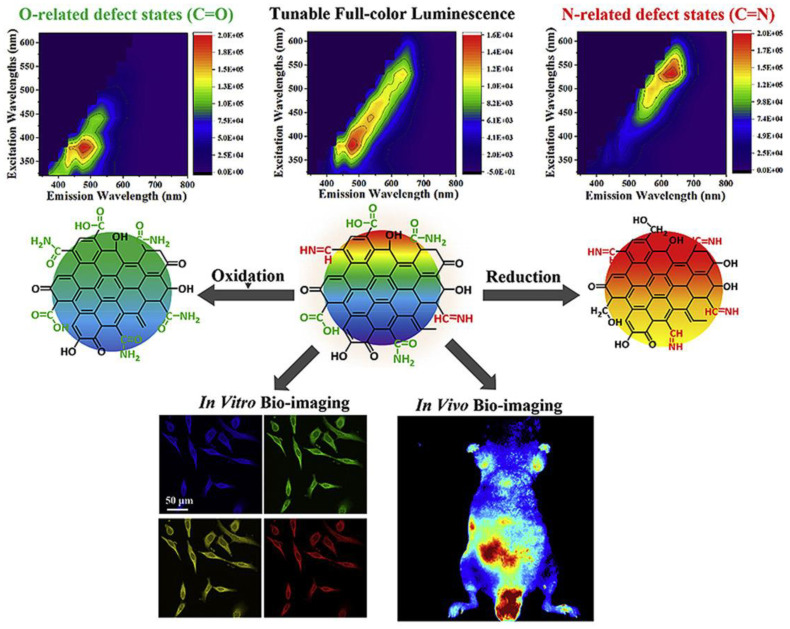
CNDs with tunable full-color luminescence for bioimaging in live cells and in mice. Adapted with permission from Reference [90]. Copyright 2020 Elsevier.

Tunable full-color luminescence—mechanism and bioimaging: Despite any research regarding CNDs and optical imaging, it is still a challenge to obtain CNDs capable of fluorescence emission covering the entire visible region. This is due to CNDs exhibiting different emission colors upon excitation with different wavelengths, with varying intensity. Here, Wang et al. have synthesized a CND type covering nearly the entire visible spectrum region (400–700 nm) [90]. Oxidation-state and reduction-state CNDs (o-CNDs and r-CNDs) were also synthesized in order to study the role of surface chemical states in determining the shifting of photoluminescence. Structural, spectroscopic, and biocompatibility analyses indicate these CNDs are favorable fluorescent probes [90]. Pristine state CNDs in this study were prepared by using a solvothermal approach that involved dissolving citric acid and urea in formamide and obtaining dark red powder by lyophilization. O-CNDs and r-CNDs were obtained by adding HNO_3_ or NaBH_4_ into CND aqueous dispersions. An absorption spectrum of the pristine state CNDs outlines a broader absorption from UV to the almost entirely visible range [90]. The fluorescence intensity of these CNDs remained comparable under different excitation wavelengths, a feature not commonly seen on synthesized CNDs. For comparison, Wang’s group synthesized blue emission CNDs, which exhibited absorption mainly in UV light. By changing the excitation wavelength from 340 to 500 nm, the emission peak shifts with intensity changing by around 30 times. The full-color CNDs, however, showed emission intensity changes only 2.3 times when shifting the excitation wavelength from 350 to 590 nm. For practical applications, CNDs would be far more useful when one type exhibits fluorescence intensities that can stay comparable with shifting excitation wavelengths, rather than depend on tuning synthesis parameters to obtain a variety of usable CNDs. Luminescence comparisons between CNDs, o-CNDs, and r-CNDs followed. Significant blue and red-shifted emissions were observed in o and r-CNDs. The functional groups and surface chemical states of all three CND types were investigated by using XPS survey spectra. While all types contain the same elements, o-CNDs lacked the C=N/C=O peak, and r-CNDs did not exhibit the COOH peak. O-CNDs and r-CNDs showed significant increases in the peaks that the other lacked. AFM topography also revealed that no significant change in the particle sizes of all three CND types occurred. This observation suggests that optical behaviors in all three types are a result of change in surface state and not size. Wang’s group proposes that during the solvothermal reaction, citric acid and urea promote reactions such as dehydration and aromatization that constitute the CNDs. The broad visible spectrum range and full-color luminescence in the pristine CNDs are provided by a variety of functional groups, such as COOH, C-OH, -CONH_2_, etc. The oxygen and nitrogen doping occurring during the synthesis of o-CNDs and r-CNDs gives rise to defect states responsible for shifting PL bands [90]. Lastly, Wang et al. evaluated CND bioimaging applications and cytotoxic effects. A549 and HeLa cells were incubated with varying CND (pristine) concentrations at different time points and exhibited viabilities above 85%. Live ells also showed bright fluorescent emissions of varying colors, indicating potential multicolor bioimaging. Nude mice were also injected subcutaneously and intravenously with CNDs. Strong fluorescence was observed in both cases, with intravenous injection showing reliable red fluorescence capable of penetrating the skin. No acute toxic response was detected in the mice at any time point. Histological studies revealed no apparent damage or inflammation in any excised organs between control and CND-treated mice [90]. These studies confirmed that the CNDs with full-color luminescence have the potential to be used in in vitro and in vivo studies (Figure 13).

### 7.2. In Vivo

Studies aiming to characterize the biodistribution of CNDs in organisms have provided evidence of rapid excretion rates in some mammals. For instance, in their study regarding N,S-co-doped CNDs as tools for photothermal therapy, Bao et al. injected twenty mice with CNDs intravenously. After the excision of significant organs, it was determined that CNDs were mostly localized in the liver and kidneys by analyzing near-infrared (NIR) fluorescent signals. After around 24 h, the signal was no longer present [70]. Strong signals were also observed 30 min post-injection, further confirming the rapid exit of CNDs. Bao et al. also observed stronger NIR fluorescent signals in areas with tumors in contrast to other tissues hours post-injection [70]. This revelation placed CNDs as potential therapeutic tools in tumor therapy.

Additionally, CNDs from aralia petals were mixed with water (1.6 mg/mL) for 1 h, and carp was put in this water. After this incubation time, carp were rinsed, and subsequent imaging showed fluorescence under 470 nm excitation [47]. Zebrafish were fed chitosan-derived CNDs, which showed time-dependent green fluorescence in the digestive system.

## 8. Biosensing

Sensing metal ions and macromolecules in biological systems needs to be developed to provide efficient, reliable, and affordable detection. The relative ease of preparing fluorescent CNDs and photostability allows for their use as economical alternatives in sensing crucial biomarkers [91,92,93,94,95,96,97,98,99,100].

### 8.1. Biosensors for Macromolecule Detection

#### 8.1.1. ROS Probe and Human Immunoglobulin Detection

Green-luminescent CNDs have been used to probe ROS such as ONOO− with high sensitivity in HeLa cells exposed for 4 h [49]. The CL intensity of these CNDS increased in a linear fashion with ONOO− concentrations from 1.0 to 100 µM in vitro [49]. The PL of CNDs was applied to the development of an immunoassay, which could detect an important immune macromolecule, human immunoglobulin (IgG) [20]. Zhu et al. hydrothermally treated CA, carboxylated the solution, and supplemented it with goat antihuman IgG (gIgG). This resulted in gIgG-CNDs that had PL intensity that was amplified when it bound IgG by a decrease in surface defects. Zhao et al. used mouse anti-human IgG (mIgG)-treated CNDs to signal the reversal of PL quenching by human IgG binding. The concentration of IgG could be calculated as a function of the PL intensity that was quenched or recovered, respectively.

#### 8.1.2. DNA Detection

One of the major macromolecules in biology, DNA, has also been identified by using rCNDs prepared by Peng et al. This was achieved by oxymercuration–demercuration, using NaBH_4_, which reduced CNDs to produce rCNDs [20]. These bonded to single-stranded DNA (ssDNA), which created a ssDNA-rCNDs probe that was hybridized when in contact with target double-stranded DNA (dsDNA). The quenching of PL by GO or CNT binding was recovered by dsDNA-rCNDs, allowing DNA detection from 6.7 to 46.0 nM. Another important molecule for CNDs detection is glutathione (GSH), a tripeptide antioxidant. Shi et al. used a CNDs-based nanosensor for GSH detection among cysteine and homocysteine [20]. The quenching of PL by gold nanoparticles (AuNPs) caused a color change, but GSH presence sterically prevented this interaction.

#### 8.1.3. Detection for the Neurotransmitter Acetylcholine, Glucose, and Other Macromolecules

CNDs on RGO could detect the neurotransmitter acetylcholine after conversion to choline, which results in ROS-mediated PL quenching. RGO-CNDs were also able to selectively detect glucose with glucose oxidase (GOx) and 10 mM bovine serum albumin in solution, serum, and saliva with a linear range of 1–60 mM. These CNDs could also detect H_2_O_2_ was due to ROS interactions that quench the PL [5].

Huang et al. were able to develop Eu^3+^-CNDs for the detection of phosphate-containing metabolites such as ATP. This probe could be turned “on” by the presence of Pi, which fragments CNDs aggregates in a selective manner that induces PL. Qiu et al. also developed a similar CNDs that was applied to detect molecules such as ATP. The PL emitting CNDs were synthesized by using CA and glutathione (GSH), an antioxidant species. Their PL was recovered by ATP after quenching with Fe^3+^, and this allowed for the successful probing of phosphate-containing molecules in human blood serum and cellular material [20].

Qiu et al. also created a glucose nanodetector that was based on the PL quenching of anionic CNDs by electron transfer for cationic boronic acid–substituted bipyridinium salt (BBV) [20]. Glucose barred this transfer by neutralizing BBV charge and recovering PL from those CNDs that had already been quenched. Thus, glucose concentrations from 1 to 60 mM could be measured linearly according to PL intensity. Qu et al. modified this approach by building “off” sensors for cis-glucose that would quench PL of CNDs passivated with 3-Aminobenzeneboronic acid [20]. Other groups such as Shen et al. enhanced the detection range and sensitivity of these CND-based glucose detectors [20].

The homeostatic balance of dopamine is disrupted in neurological diseases such as Alzheimer’s disease or Parkinson’s disease. Most current methods of detecting this neurotransmitter are not economical, which prompted the development of CND-based dopamine sensors. These CNDs underwent surface electron transfer in the presence of dopamine, showing strong and selective fluorescence in neutral solution [101].

CNDs sensitive to Ag^+^ were used to develop an array that detected biothiols, which are central players in antioxidant defense, immune function, and many catalytic reactions. The fluorescence of these CNDs was quenched by Ag^+^ binding and recovered upon biothiol binding and subsequent removal of Ag^+^. This array was developed by using the unique binding abilities of the CNDs, which allowed detection of many biothiols, including glutathione (GSH), cysteamine, and mercaptoethanol, at a range >10 µM [102].

### 8.2. Biosensors for Metals and Ions Detection

#### 8.2.1. Mercury (II) Ion (Hg^2+^) Detection

The previous analysis of the PL properties of CNDs has paved the way for many biochemical and environmental sensing applications. The importance of developing methods to detect mercury (II) ion (Hg^2+^) is evident from its environmental abundance and effects on the CNS and endocrine system. Early design of CNDs allowed Hg^2+^ did not allow for sensing in anything but water, which excluded any buffers or electrolytes [43,103,104,105,106,107,108,109,110]. Hg^2+^ nanosensing was further developed by Gao et al. with a nanohybrid of red-emission QDs and blue-emission CNDs. The altered QDs had carboxyl-methyldithiocarbamate, which is a strong chelating agent and can quench their red PL emission [20]. CNDs had a constant blue emission, which resulted in a red-to-blue color gradient that could be used to detect Hg^2+^ up to 0.1 µM. Methyl-Hg could also be detected by CNDs in a PEG-ylated CND developed by Costas-Mora. PL of these CNDs was quenched only by hydrophobic Methyl-Hg and could detect its concentration down to 5.9 nM [20]. This method gave results that reflected the sensitive and concentration-dependent design of CNDs as mercury-based sensors.

These current methods to detect Hg^2+^ are costly and specialized, creating the need for economic alternatives. EDTA-CNDs detected Hg^2+^ down to 4.2 nM, but further efforts created sweet-potato-derived CNDs that had a Hg^2+^ detection limit of 1 nM [111]. The PL intensity decreased due to quenching by Hg^2+,^ possibly by electron transfer. Fluorescence was restored by adding Cys, a chelator that removed Hg^2+^ from the CND surface. Green-synthesized N,S-CNDs were used for detecting Hg^2+^ in vivo [50]. PEG_200_-CNDs were used as a highly sensitive nanosensor of Hg^2+^ due to the quenching effect of Hg^2+^ and Cu^2+^ had on PL of CNDs. These ions complex with functional groups on the modified CND surface, allowing their use as a Hg^2+^ sensor that was stable, rapid, widely applicable, and reversible. A “turn-on” sensor was developed to detect Hg^2+^ by using functionalized bis(dithiocarbamato)copper(II) on amine-containing carbon nanodots (CuDTC_2_-CNDs). The CuDTC_2_ complex interacted with Hg^2+^ and produced fluorescence in a highly sensitive and dose-dependent manner (40 nm–1 µM) [112]. These sensors were able to detect Hg^2+^ in lake water, tap water, and urine samples.

#### 8.2.2. Ion Al^3^ Detection

The ion Al^3+^ is a crucial player in many biological events, and its imbalance can cause damage to kidneys, bones, and the nervous system. The CND–dopamine system mentioned earlier was used as a selective detector for Al^3+^ showed recovery of fluorescence upon its interaction. The relative intensity of fluorescence increased linearly with the Al^3+^ concentration (4–40 µM). This nanosensor was selective in the presence of various other components, as it detected both molecules in human serum, urine, and food [101].

#### 8.2.3. Copper (Cu) Detection

The role of copper (Cu) is essential in the enzymatic activity of SOD and cellular signaling, and its imbalance is linked to neurological disorders such as Alzheimer’s disease. The interaction between ROS and Cu^2+^ causes the abnormal folding of proteins, which creates a need for sensitive and efficient nanosensors for this ion [89]. Chi et al. were able to create Cu^2+^ probes by passivating CNDs with polyethylenimine (BPEI) and citric acid (CA) [20]. These surface functional groups captured Cu^2+^ and quenched the PL, being able to detect the ion from 6 to 1100 nM, with high precision [20]. These sensors were also applied in living cells by Qu et al., being able to select Cu^2+^ among other components and transfer their charge to this ion only [20]. This was read as a decrease in PL, which was used to detect L-cysteine and Cu^2+^ as they had opposing effects on the PL of these modified CNDs [20]. “Turn-on” assays for Cu^2+^ detection were carried out by o-phenylenediamine (OPD) CNDs that changed color from yellow to orange and increased PL intensity due to Cu(OPD)_2_ complex formation. The OPD-CNDs detected cellular Cu^2+^ ions linearly from 2 to 80 nM [5,89]. Green fluorescent CNDs (G-CNDs) from leeks were tolerant to a wide range of pH, with sensitive detection even in the presence of external cation (pH 3.5–10.0). Similarly synthesized blue fluorescent CNDs (B-CNDs) detected pH and Cu^2+^ (0.01–10.00 µM Cu^2+^) in a selective linear fashion in vitro [25].

#### 8.2.4. Concentration of H^+^ Detection

The concentration of H^+^ in organisms is essential for metabolism, and imbalances can lead to significant cardiac, neurological and other disorders [25]. The effect of pH on PL of CNDs was emphasized in the prior discussion, which indicated their use as pH sensors in solution. The mode of synthesis imparts sensing capabilities, with some CNDs decreasing PL in a linear fashion as the pH increases. This method used a fluorescein isothiocyanate dye (FITC) that was used to treat CNDs, so their emission increased from pH 5 to 8 [20]. N-CNDs showed a significant decrease of PL intensity in acidic solution, which then increased linearly with the pH. The most sensitive change in PL was observed between pH 7 and 9, which implies their use in detection in biological systems [10]. Hydrophilic CNDs showed decreased fluorescence at both extremes of pH values, which is hypothesized to be due to amine and carboxylic acid functional groups undergoing protonation or deprotonation. This is supported by the reversal of fluorescence when the pH of the solution was returned to its original value [27]. CNDs showed reversible absorption of UV–Vis wavelengths and PL properties at a broad pH range (3–13) due to differential surface behavior. Acidic solution caused self-directed aggregation and oxidation of CNDs, indicating the surface changes led to new PL intensities. Inversely, CNDs in basic solution underwent tautomerization and gradual hydrogenation/deoxygenation, which altered the PL behavior [113]. HeLa cells were incubated with B-CNDs (0.6 mg/mL) to study pH-dependent fluorescence, using confocal microscopy. Results showed strong blue fluorescence starting at pH 10.0 and reduction when the pH was decreased, which indicates the use of B-CNDs for imaging in live cells [25].

#### 8.2.5. Detection of Fe (III) and the Underlying Mechanisms

Another ion that can quench the PL of CNDs is Fe^3+^ [42,114,115,116,117,118,119,120], but this is highly susceptible to interference by other metal ions. To improve the efficiency of Fe^3+^ biosensors, Qian et al. found that L-lysine recovers the PL quenched by Fe^3+^. Yang et al. also found Fe^2+^ and H_2_O_2_ worked similarly and could be used to detect either chemical [42]. The quenching effect was either due to excited-state electron transfer between CNDs and Fe^3+^, formation of carboxylic acid–Fe^3+^ complexes, or ROS formation. Cytochrome C was also found to quench CNDs due to its Fe^3+^ ions interacting with CNDs hydroxyl groups, which was completely reversed by trypsin. Thus, Cytochrome C–CNDs complexes could be used in biological systems for the detection of trypsin as well.

**Figure 14:**
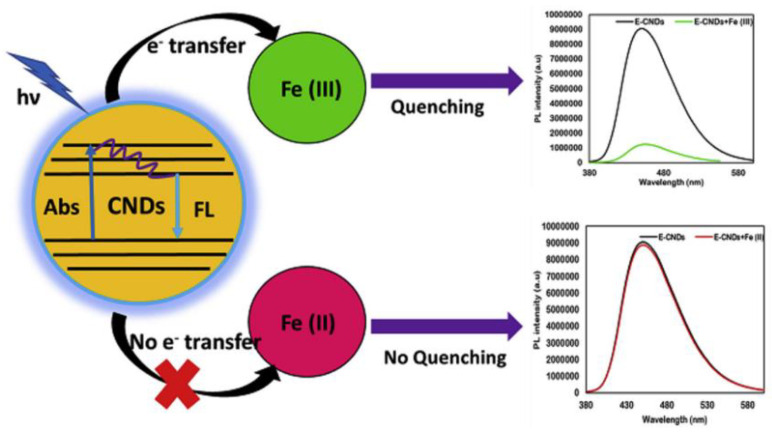
Detection of Fe (III) Ions via CNDs fluorescence quenching reaction. Adapted with permission from Reference [42]. Copyright 2020 Elsevier.

CNDs have also garnered attention in the biosensing of metal ions. As key parts of biological processes, the monitoring of metal ions is a chief concern in biomedical applications. Iron, for instance, is important for body temperature regulation, transport of oxygen in blood, and brain development of newborns. Current methods of detection rely on complex instruments and convoluted preparation of samples. Use of fluorescent materials exhibit great potential but are hampered by irreversible photobleaching and other limitations [42]. Nanomaterials have been deemed potential replacement options but have limited use with cells due to cytotoxicity. Despite recent advances in the field of biosensing, it remains a challenge to effectively detect Fe (III) ions. Arvapalli’s group have synthesized and characterized a type of CNDs with potential as a sensing platform for Fe (III) ions. In their study, two types of fluorescent CNDs were synthesized with a single step microwave process. E-CNDs were synthesized from ethylene diamine (64% QY), and U-CNDs from urea (8.4%). Characterization revealed a few key differences between the two types. E-CNDs showed a higher presence of nitrogen content. Both showed C-O-C, C-C, and O-C=O functional groups, with E-CNDs additionally exhibiting C-O groups. E-CNDs displayed a surface charge of around −7.32, whereas U-CNDs had a more negative charge of around −38.5. This was attributed to U-CNDs retaining more COO- groups and E-CNDs having a higher count of positive amine groups. Regardless, the hydrophilic nature of the CND types is largely due to amino, carboxyl, and hydroxyl groups. Other key similarities included strong emission peaks at ~450 nm and blue light emission under UV irradiation. Excitation-dependent emission wavelengths, however, were observed to be at different wavelengths from 330 to 450 nm [42]. Both E-CNDs and U-CNDs demonstrated rapid fluorescence quenching under 30 s with the addition of Fe (III) ions. Quenching was also observed to increase with increasing dosage of Fe (III) ions, revealing a dose-dependent response. Limits of detection were also established as 18 nm and 30 nm for E and U-CNDs, respectively, indicating these CND types were good performers in sensing iron ions. No significant decrease instability was also observed in a period of 50 days. Using 50 µM concentrations, the selectivity towards other ions was also analyzed (Ag^+^, Co^2+^, Mg^2+^, etc.). No significant quenching was observed in the presence of other metal ions, but quenching was observed when mixing in other ions with Fe (III) ions and exposing them with either CND type. The quenching efficiency of E-CNDs was ~87% and ~70% for U-CNDs. These results indicated both CND types were highly selective in sensing Fe (III) ions. Further testing included assessing the detection of iron in tap water and human serum. E-CNDs detected 3.8 µM and U-CNDs 1 µM of iron content in tap water (27.8 µM and 24.6 µM for human serum). In living EA.hy926 cells, both CND types, showed localization with blue fluorescence. Significant quenching of fluorescence was observed in cells incubated with both CND types and Fe (III) ions after 30 min, with near-complete quenching noted after an hour. Next, Arvapalli’s group investigated possible quenching mechanisms between Fe (III) ions and both CND types. Following a Stern–Volmer plot, the quenching constant (K_sv_) in low concentration linear range was higher in E-CNDs. The larger constant denotes faster kinetics of CND and Fe (III) association. This was further verified by using cyclic voltammograms (CV). The redox peaks of Fe (III) ions were observed to decrease with the increasing concentration of CNDs, indicating the formation of CND-Fe (III) complexes. E-CNDs exhibited more substantial decreases in peak current with lower concentrations than U-CNDs, denoting kinetically faster quenching reactions with Fe (III) ions [42]. These results also gave rise to the notion that electron transfer occurs from CNDs to Fe (III) ions, resulting in fluorescence quenching. Though both CND types are valuable replacement options for sensing Fe (III) ions, E-CNDs may be the more efficient option. Collectively, this study shows a potentially invaluable new tool for the selective sensing of Fe (III) ions (Figure 14).

## 9. Biocompatibility and Potential Toxicity of CNDs

Most studies regarding the biocompatibility of CNDs have been performed in vitro. Qi et al. applied CNDs to VERO cells, which engulfed them through endocytosis and showed low toxicity as measured by MTT assay [20]. Cell viability in LLC-PK1 cells was measured after treatment with coffee-ground-synthesized CNDs up to 2.4 mg/mL. Results showed 95% or greater viability up to 1.8 mg/mL CNDs, which further supporting their biocompatibility [24]. CNDs from citric acid were incubated with HUVECs presented low toxicity as indicated by the MTT assay, which can further their potential in detecting dopamine in the nervous system [101]. As previously mentioned, researchers can tune the surface functional groups in CNDs. This allows the scientist to tailor the reactivity of the nanoparticle for specific uses. Recently, studies have attempted to characterize the interactions of CNDs with different cell types and their effects on viability. As part of a study of antioxidant properties in N,S-co-doped CNDs, Zhang et al. performed MTT assays with concentrations of up to 0.10 mg/mL for 24 h and found no significant effects on EA.hy926 cells [6]. Similarly, Ji et al. performed MTT assays on EA.hy926 and A549 cells treated with N,S-co-doped CNDs with concentrations between 0.1 and 0.8 mg/mL. Both cell lines demonstrated adverse effects on cell viability with increasing concentrations of these CNDs, with EA.hy926 cells showing more sensitivity. This decrease in viability has been attributed to the dose-dependent increase in ROS generation of the N,S-co-doped CNDs [121]. Given that most CND studies on cell viability denote low cytotoxicity, these twin studies accentuate that adverse effect caused by CNDs largely depends on their surface functional groups, as well as the working range of concentration.

The available literature regarding the biocompatibility of CNDs in vivo applications is not extensive. However, some advances have been made in recent years. Studies addressing the long-term biodistribution of CNDs in mice showed they concentrated in the liver, lung, spleen, kidney, and tumor sites. However, histological and biochemical examinations showed no toxic effects [20]. In vivo tests revealed 8 and 40 mg/kg body weight doses of EDA-CNDs had low toxicity and no significant impairments in the activity of mice. Imaging revealed the accumulation of CNDs in vivo was low and did not cause observable organ damage. Dissection of the kidney and liver showed intravenous administration of CNDs results in significant excretion via urine. Unless CNDs were functionalized to target specific cells, organs did not show any PL after treatment, indicating the relative absence of CNDs [47]. Mice were given CNDs (~23 mg/kg) via subcutaneous injection and did not show statistically significant difference in fluorescence or body weight between control and experimental groups at any point during a three-week period. Toxicity studies of the heart, lung, liver, spleen, and kidneys showed no edema, inflammation, or signs of pathology when compared to the control mice [88]. Bao et al. and their study on in vivo theranostics with a mice model also demonstrate near-total excretion of intravenously injected CNDs after 24 h, giving rise to the notion that CNDs are too rapidly removed from the body to cause harmful effect [70].

## 10. Conclusion and Future Studies

In summary, this review article outlined a modern understanding of CNDs and their applications. Popular synthesis techniques were discussed in detail, covering top-down and bottom-up approaches, as well as advances in green synthesis. This culminates in a vast number of synthesis routes, proving advantageous for many scientific studies, as CNDs tailored for specific tasks can be more readily fabricated. Previously, several CND synthesis methods were limited due to unreliable quantum yields of less than 20%. In recent years, the synthesis of CNDs has seen a notable boost in quantum yield, both in top-down and bottom-up approaches. This review also included a comprehensive discussion over the many factors that play into the PL properties of CNDs, including size, precursors, surface functional groups, and even time.

We also discussed the cruciality of surface passivation in CND biodistribution. In vitro studies indicate that certain CND types can localize more effectively in the cytoplasm or mitochondria, whereas others can penetrate the nucleus and interact with genetic material. Few works in the literature exist that analyze CND uptake and exit routes in cells. Several studies often mention that endocytosis is a likely uptake route in cells, but few studies address this notion with experiments and data. A study designed to compare the localization of different CND types would provide essential information in selecting the most appropriate CNDs for specific biomedical tasks.

Recent advances regarding CNDs as therapeutic agents were also discussed. CNDs have been functionalized as chemo-sensors of ions and metals, with some specific to one type of metal ion. Research also indicates that CNDs may replace fluorescent dyes as a more efficient biocompatible alternative. Advances were also discussed regarding the bioimaging of macromolecules. In vivo studies have demonstrated rapid excretion rates in mammals with intravenous CND injection, with strong localization mainly in the liver and kidneys. The currently available literature regarding in vivo studies is still extremely limited, but we have noted in this review some important research in therapy. For instance, research placed PDA-CNDs as efficient therapeutic agents in countering AKI (acute kidney injury) in mouse models, mainly due to their ability to scavenge ROS. These results open the possibility of CNDs providing a remedy against other disease states.

Subsequently, a key point of discussion in this review is recent advances regarding CND antioxidant properties. Of notable interest is the revelation that functionalization of CNDs tailors their efficiency at scavenging ROS particles (for instance, N,S-CNDs are more efficient at scavenging DPPH than superoxide anions). Interestingly, blocking key functional groups, such as carboxylic acids, limits the antioxidant potential of N,S-CNDs. This study can be furthered by blocking the same functional groups in a repertoire of CND types and analyzing antioxidation impact. With the growing number of types due to the various methods of synthesis, studies such as this would allow for better choice of CNDs in different applications. The discussed articles collectively seem to agree that the mechanism of action in ROS scavenging is due to hydrogen atom transfer (HAT). This notion is supported by electrochemical studies performed. The crucial role that ROS plays in conditions associated with inflammation has recently spawned investigations utilizing CNDs. In the case of atherosclerosis, it is widely accepted that ROS serves as a key mediator of the disease state. Therefore, we discussed valuable research that has characterized the effects of CNDs on cells involved in atherosclerosis, including macrophages and endothelial cells. This research has led to the hypothesis that CNDs directly or indirectly affect essential proteins and signaling pathways involved in the inflammatory response, such as the Nf-kB pathway, STATs, and antioxidant proteins such as Nrf2. Presently, no research has investigated this hypothesis. With the growing health concern that is atherosclerosis and cardiovascular disease, it is important to investigate this hypothesis, as CNDs proffer themselves as potential therapeutic instruments.

Despite the significant progress scientists have made with CNDs in the last five years, there is still much to learn about these nanoparticles. There is still considerable room for improvements in more accurate synthesis methods. Several biomedical fields have also been left unexplored, with research available for only a few key disease states. Swift research into developing CNDs with more controllable properties may further assist in realizing their full potential as biomedical devices.

## Data Availability

The data used to support the findings of this study are available from the corresponding author upon request.
